# Supplementary material for: Streamlined single-cell proteomics by an integrated microfluidic chip and data-independent acquisition mass spectrometry
Source: Nat Commun. 2022 Jan 10;13:37. doi: 10.1038/s41467-021-27778-4 (PMC8748772; doi:10.1038/s41467-021-27778-4)
Supplement: Supplementary file 1 — Supplementary Information [file 41467_2021_27778_MOESM1_ESM.pdf]

Supplementary Information

Streamlined single-cell proteomics by an integrated microfluidic chip and data-independent acquisition mass spectrometry

Sofani Tafesse Gebreyesus[1,2,3,†], Asad Ali Siyal[1,4,5,†], Reta Birhanu Kitata[1], Eric Sheng-Wen Chen[1], Bayarmaa Enkhbayar[4,6], Takashi Angata[6], Kuo-I Lin[7], Yu-Ju Chen[1,3,4,8,*] and Hsiung-Lin Tu[1,2,4,8,*]

1. Institute of Chemistry, Academia Sinica, Taipei 11529, Taiwan
2. Nano Science and Technology Program, Taiwan International Graduate Program, Academia Sinica, Taipei 11529, Taiwan
3. Department of Chemistry, National Taiwan University, Taipei 10617, Taiwan
4. Chemical Biology and Molecular Biophysics Program, Taiwan International Graduate Program, Academia Sinica, Taipei 11529, Taiwan
5. Department of Chemistry, National Tsing Hua University, Hsinchu 30013, Taiwan
6. Institute of Biological Chemistry, Academia Sinica, Taipei 11529, Taiwan
7. Genomics Research Center, Academia Sinica, Taipei 11529, Taiwan
8. Genome and Systems Biology Degree Program, Academia Sinica and National Taiwan University, Taipei 10617, Taiwan

† These authors contributed equally: S. T. Gebreyesus and A. A. Siyal

* Email: yujuchen@gate.sinica.edu.tw (Y.-J. Chen) and hltu@gate.sinica.edu.tw (H.-L. Tu)

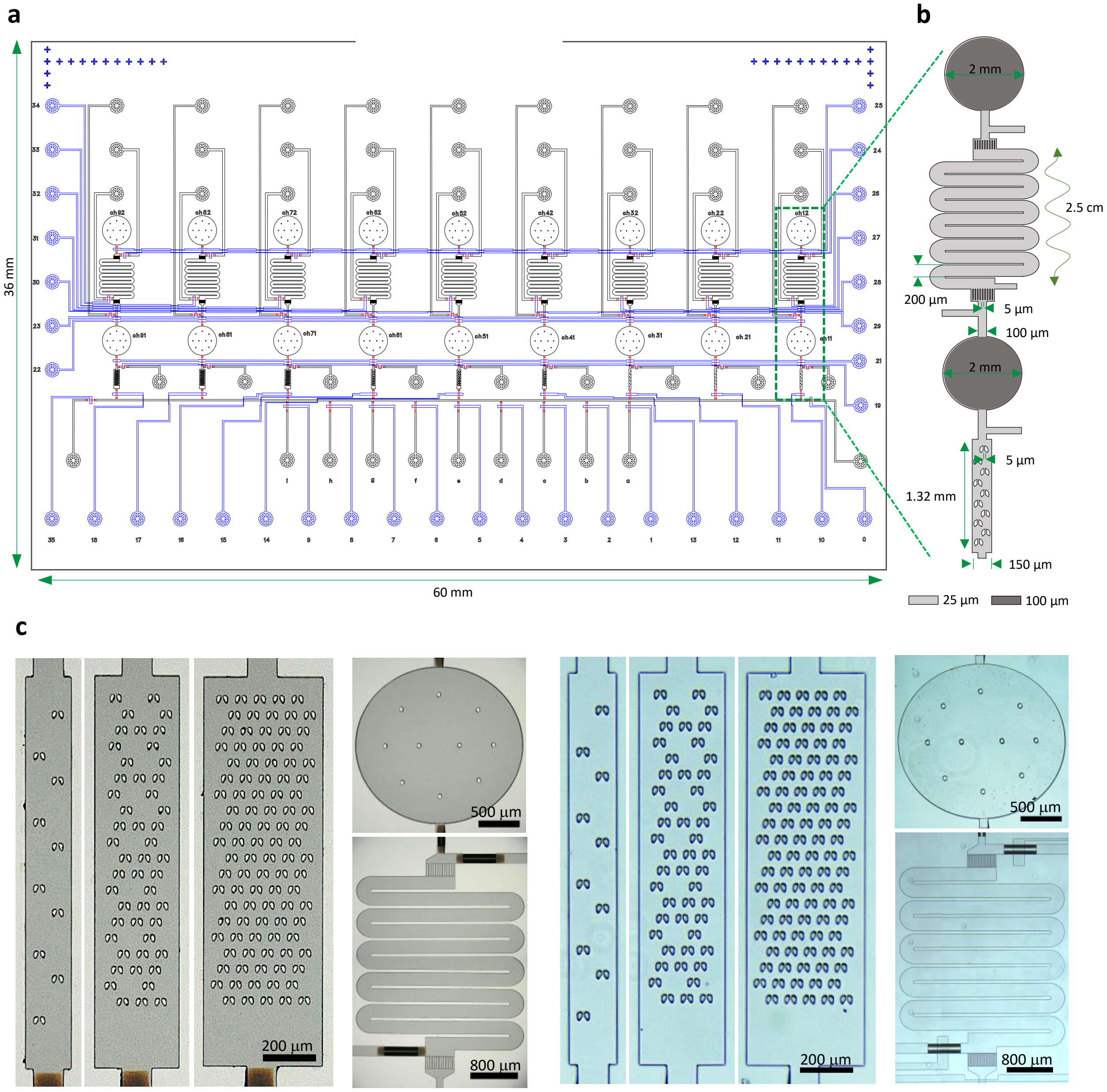

Supplementary Figure 1. The Overview of iProChip design and fabrication. (**a**) The device layout; flow layer (black and red) is a channel network containing cell/reagent inlets, cell capturing chambers, reaction vessels, solid phase extraction columns and sample collection ports. There are nine operational lines in a single device consisting of triplicates for 10, 50 and 100 cells, all sharing the same buffer and cell inlets. The control layer is shown in blue and contains 34 actuation valves. (**b**) A single operational unit showing the dimensions for main sections in iProChip. The height of the entire flow layer is 25 µm except for chambers in dark grey which is 100 µm. (**c**) Images taken from main modules, including cell capturing chambers, a reaction vessel and SPE column, on the silicon wafer mold (left panel) and corresponding PDMS replica (right panel).

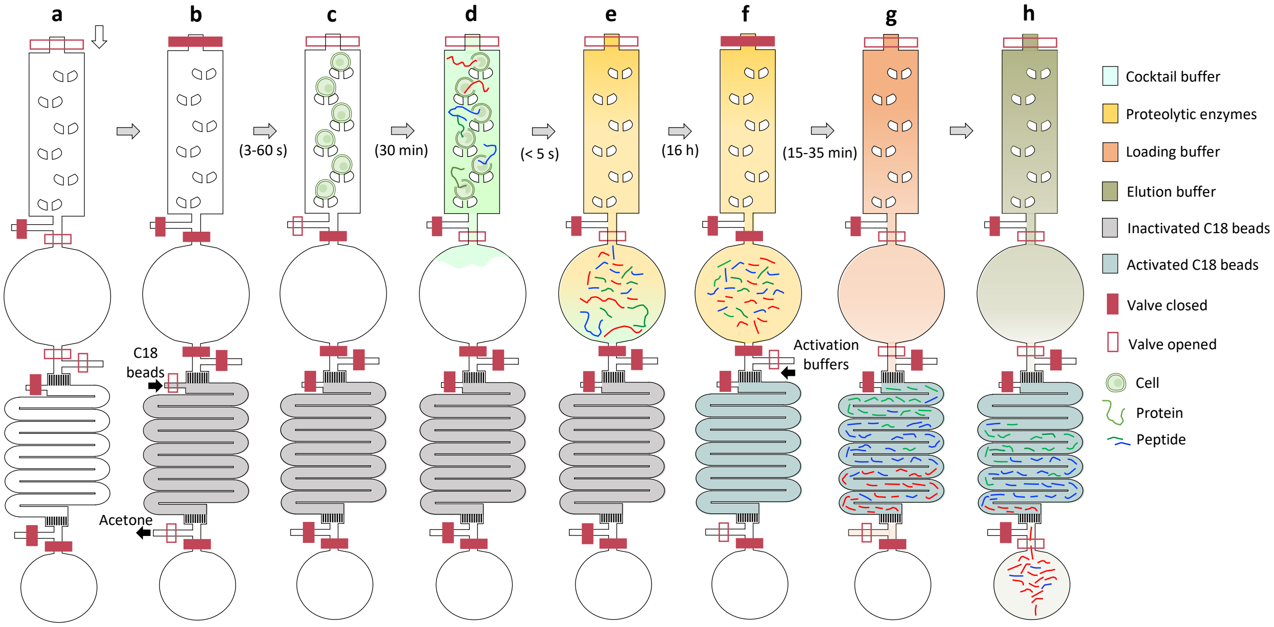

Supplementary Figure 2. The entire operating procedures of iProChip for proteomics analysis. (**a**) Coating of the entire chip (except SPE column) with 0.1% BSA. (**b**) Packing of reverse phase C18 beads to prepare SPE desalting column. (**c**) Capturing of cells. (**d**) Lysis of cells, followed by reduction (cysteine) and alkylation using a cocktail buffer. (**e**) Enzymatic digestion and acidification by formic acid. (**f**) Activation of C18 beads in SPE column with activation buffers. (**g**) Cleaning-up of digested peptides through the SPE column. (**h**) Elution and collection of peptides. It takes approximately 20 h to complete all of the steps of the operation for 9 samples.

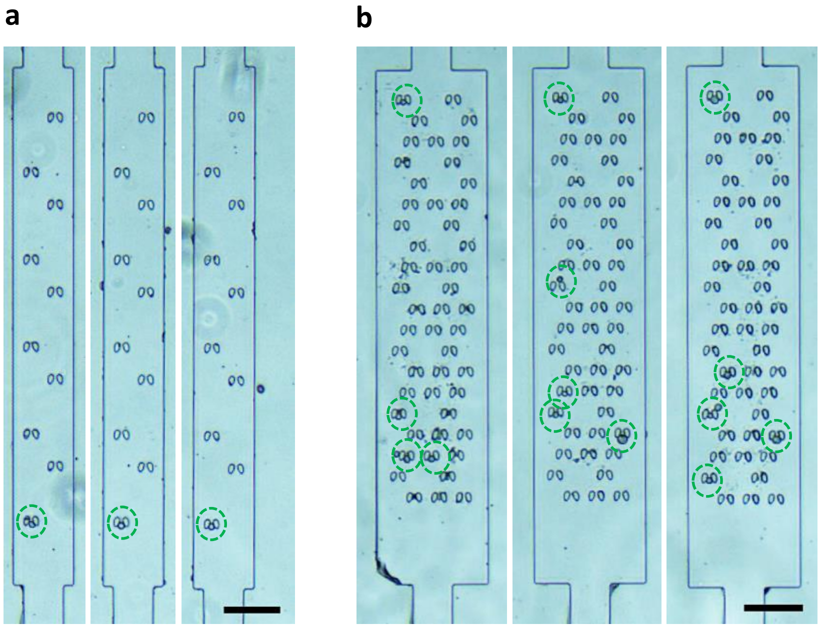

Supplementary Figure 3. Single and low numbers of cell capturing using iProChip. (**a**) Bright-field images demonstrating facile capturing of single PC-9 cell (green circle) using 10 cell capture chambers. (**b**) Bright-field images showing capturing of 5 PC-9 cells using 50 cell capture chambers. Scale bars: 150 mm. Cell density used for these experiments was kept at 25 cells/µL.

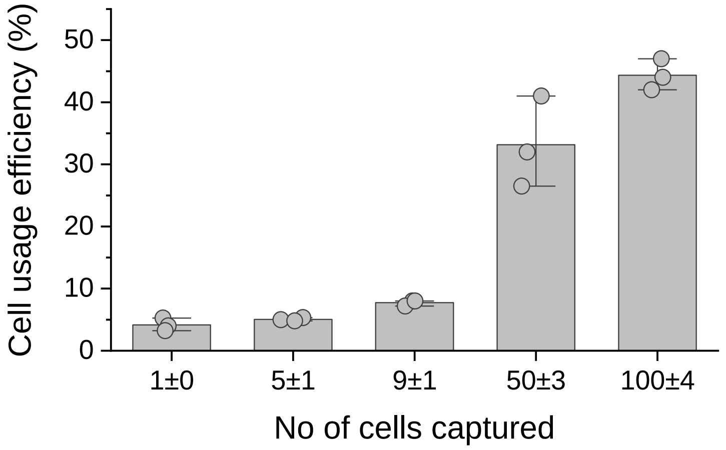

Supplementary Figure 4. The cell usage efficiency for capturing 1, 5, 10, 50 and 100 cells using iProChip as determined by using 5 or 10 µL cell solution with a density of 25 cell/µL. Data are presented as mean values ± SD (*n* = 3 independent experiments). Source data are provided as a Source Data file.

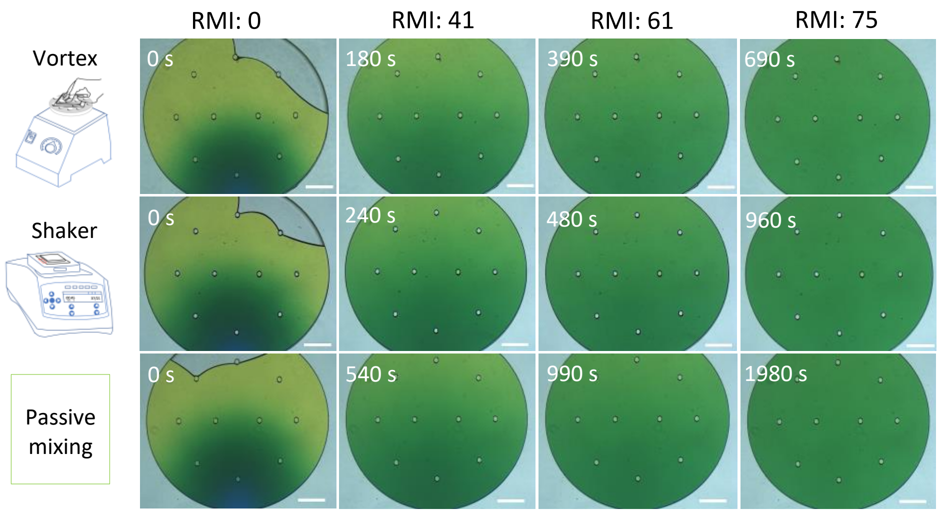

Supplementary Figure 5. Mixing performance tests between by vortex, by shaker and through diffusion. Bright-field images of reaction vessels during mixing tests by vortex (top row), by shaker (middle row) and through diffusion (bottom row), respectively. Each column shows the time point where mixing performance as determined by the calculated relative mixing index (RMI) reached the same level. Scale bars: 300 µm. Source data are provided as a Source Data file.

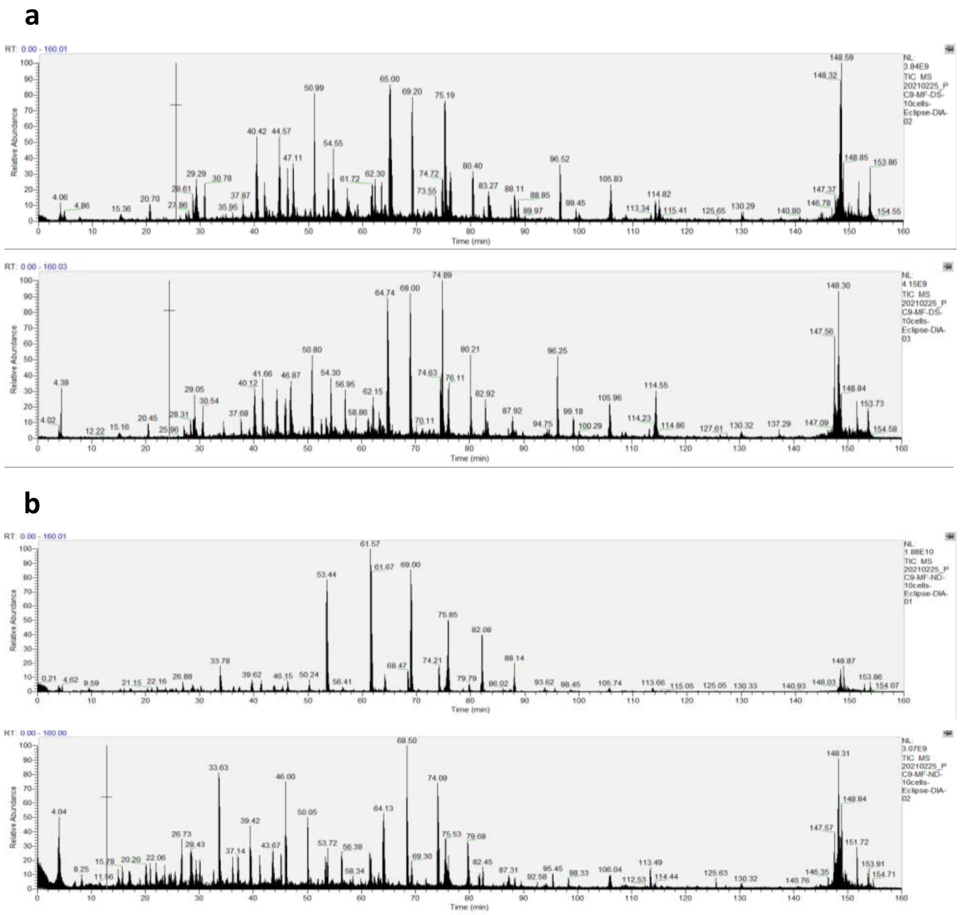

Supplementary Figure 6. Evaluation of desalting performance of SPE columns. Comparison of the total ion chromatogram (TIC) profiles of (a) desalted and (b) non-desalted 10-cell samples processed through the iProChip, respectively.

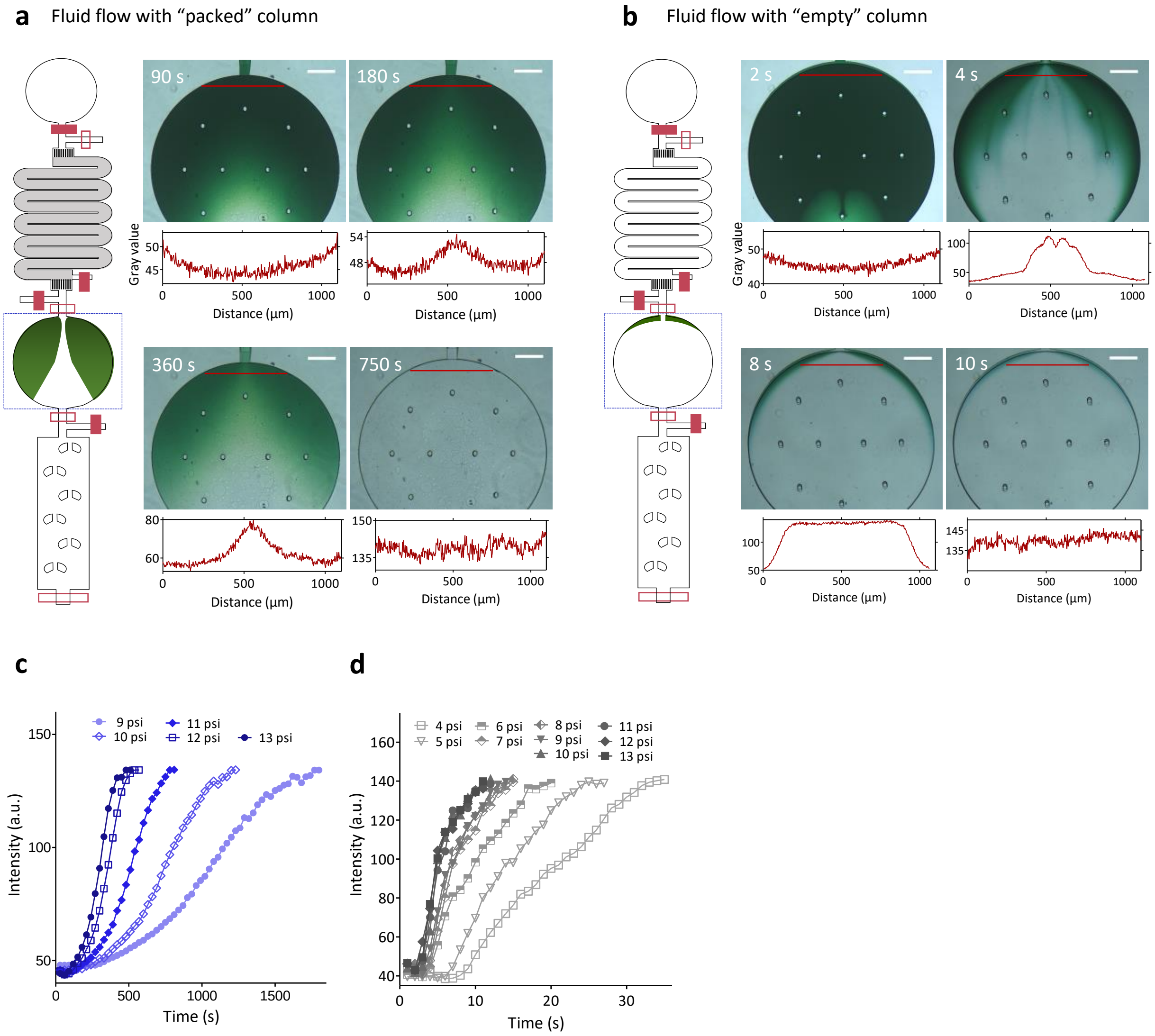

Supplementary Figure 7. Characterization of optimal flow condition in the reaction vessel. A cartoon illustration (left) and real images (right) of fluid in the reaction vessel flow through either (**a**) C18 beads-packed or (**b**) empty SPE column at flow pressure of 11 psi. Scale bars: 300 μm. Note the significantly different time scales. The complete change in color intensity within (**c**) 1500 s and (**d**) 35 s for reaction vessels when flowing into either C18 beads-packed or empty SPE columns respectively, confirming the pressure and time required for total transfer of the dye without preferential flow. Source data are provided as a Source Data file.

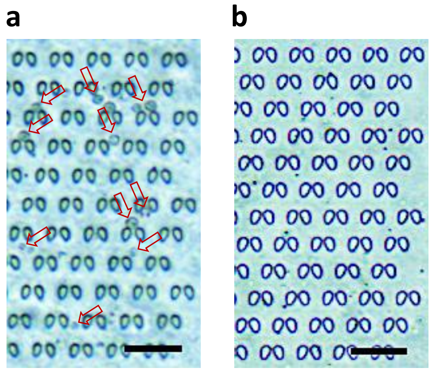

Supplementary Figure 8. Optimization of cell lysis condition. (**a**) Cell lysis under the condition of 70 °C, 10 min and 0.1% RG. Red arrows indicate un-lysed cells. (**b**) Complete cell lysis under the condition of 70 °C, 30 min and 0.3% RG. Scale bars: 200 µm.

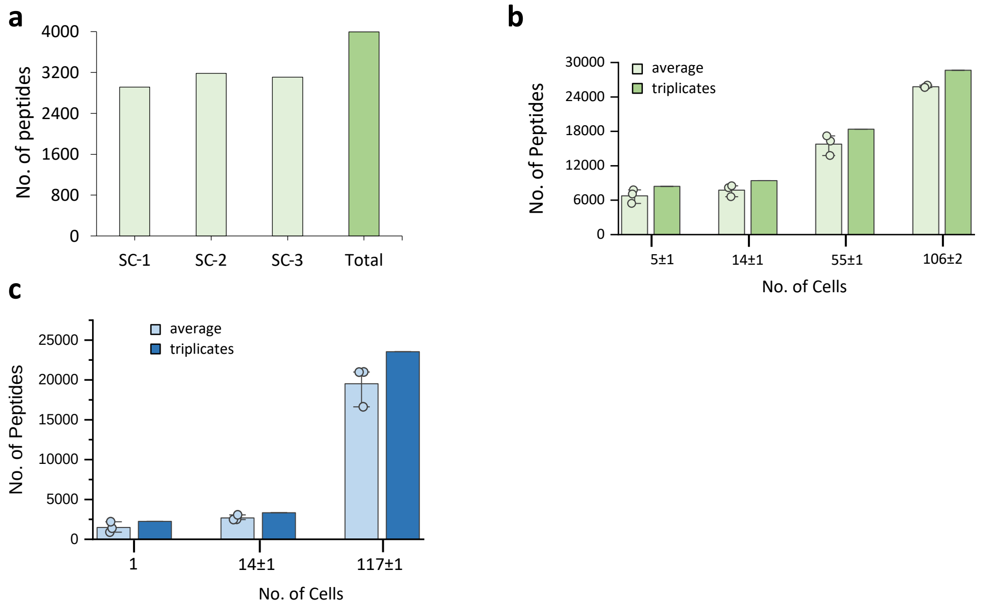

Supplementary Figure 9. Identification summary of peptides across different cell numbers. Peptide identification summary of (**a**) a single PC-9 cell, (**b**) 5-106 PC-9 cells and (**c**) 1-117 MEC-1 cells using iProChip-DIA. Data are presented as mean values ± SD (*n* = 3 independent experiment). Source data are provided as a Source Data file.

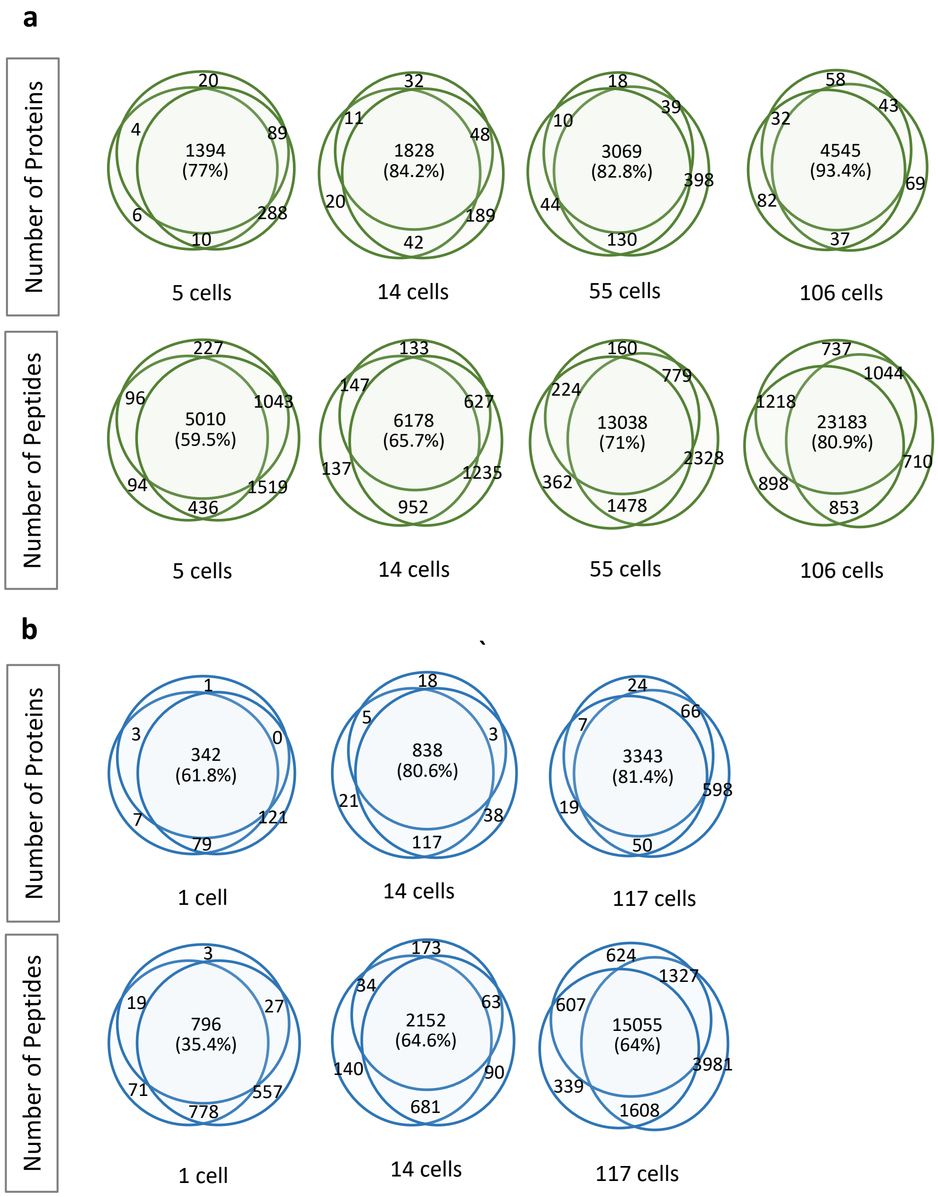

Supplementary Figure 10. Overlaps of protein groups and peptides identified in triplicate analyses of different cell numbers. (a) PC-9 cells (green) and (b) MEC-1 cells (blue). Source data are provided as a Source Data file.

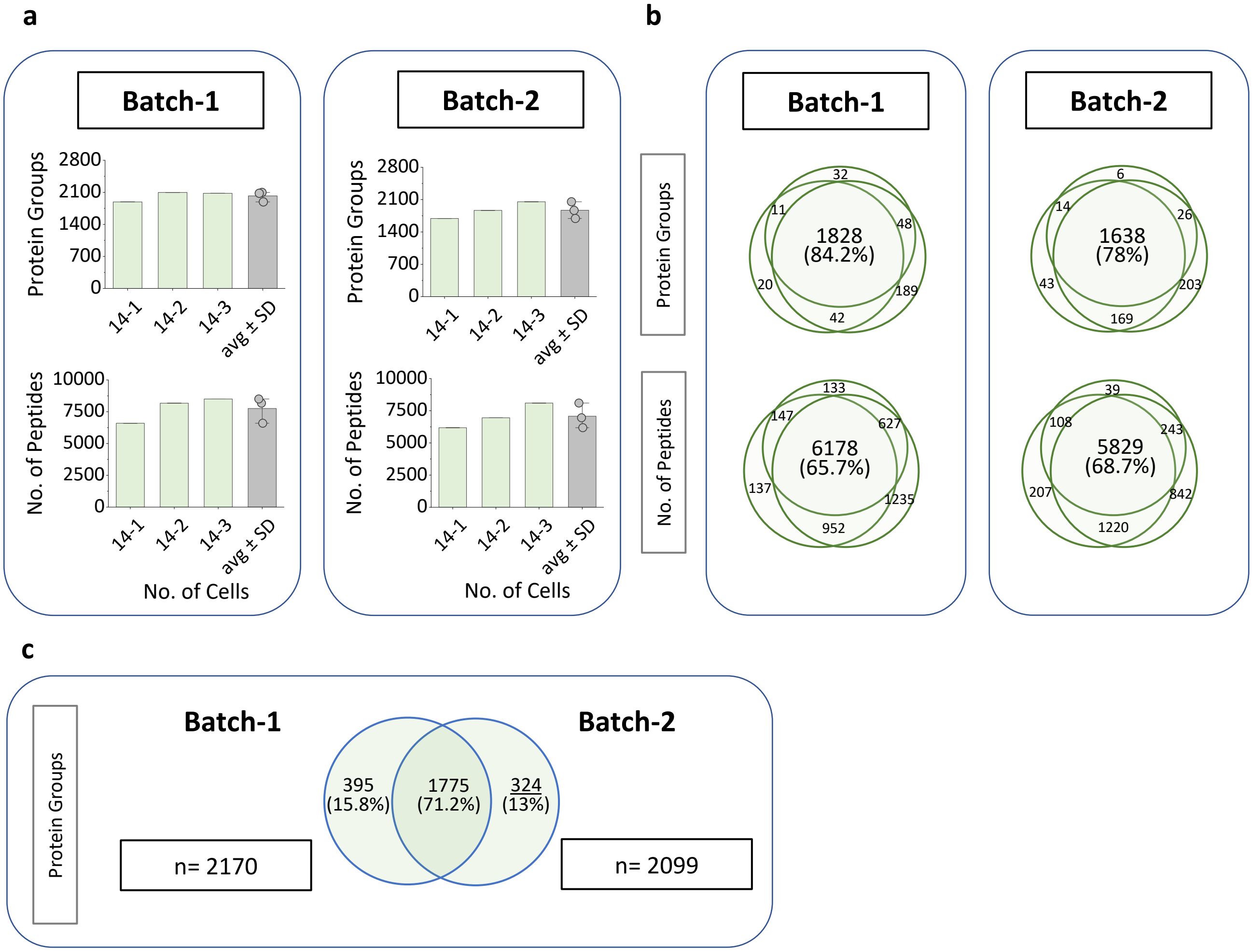

Supplementary Figure 11. Evaluation of reproducibility in triplicate analysis of 10 PC-9 cells among two experimental batches. (a) Comparison of identification of protein groups and peptides in 10-cell data from two distinct experimental batches. Data are presented as mean values ± SD (*n* = 3 independent experiments). (b) The Venn diagram classification of proteins and peptides among triplicates in both experiments. (c) Comparison of total protein groups among both batches of experiments. Source data are provided as a Source Data file.

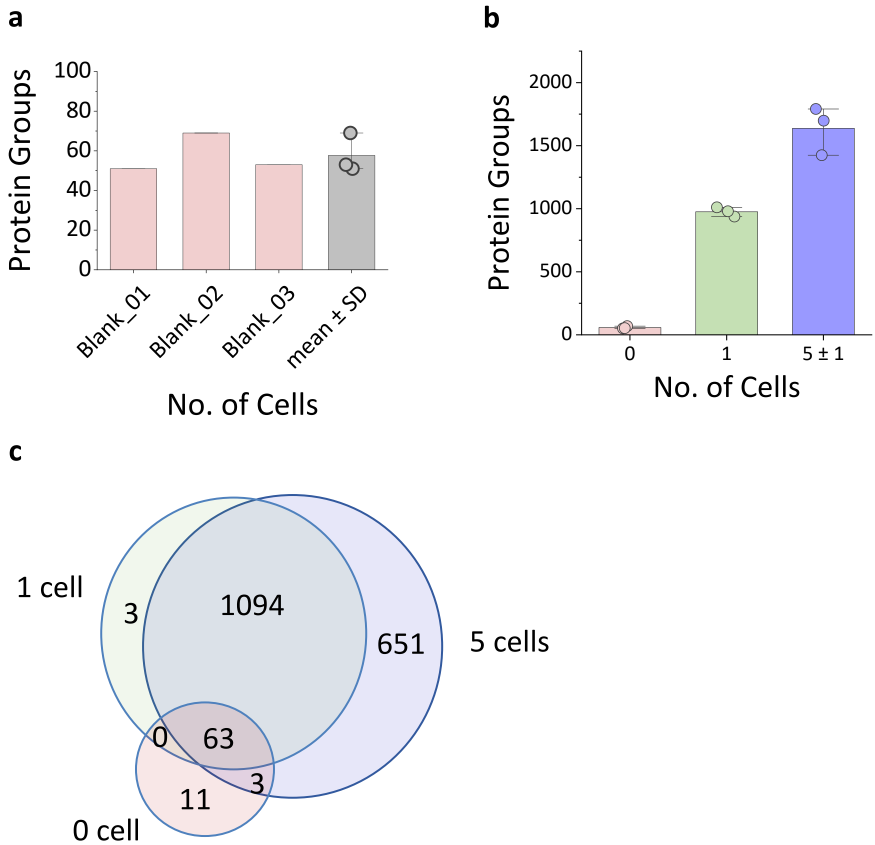

Supplementary Figure 12. Protein groups identification in triplicate analyses of blank (zero cell) samples. (a) The number of proteins identified across replicates of blank samples and the calculated average. (b) Comparison of protein identification among blank, 1-cell and 5-cells samples. Data in (a) and (b) are presented as mean values ± SD (n = 3 independent experiments). (c) Venn diagram of overlapping protein identified from blank, 1-cell and 5-cells samples. Source data are provided as a Source Data file.

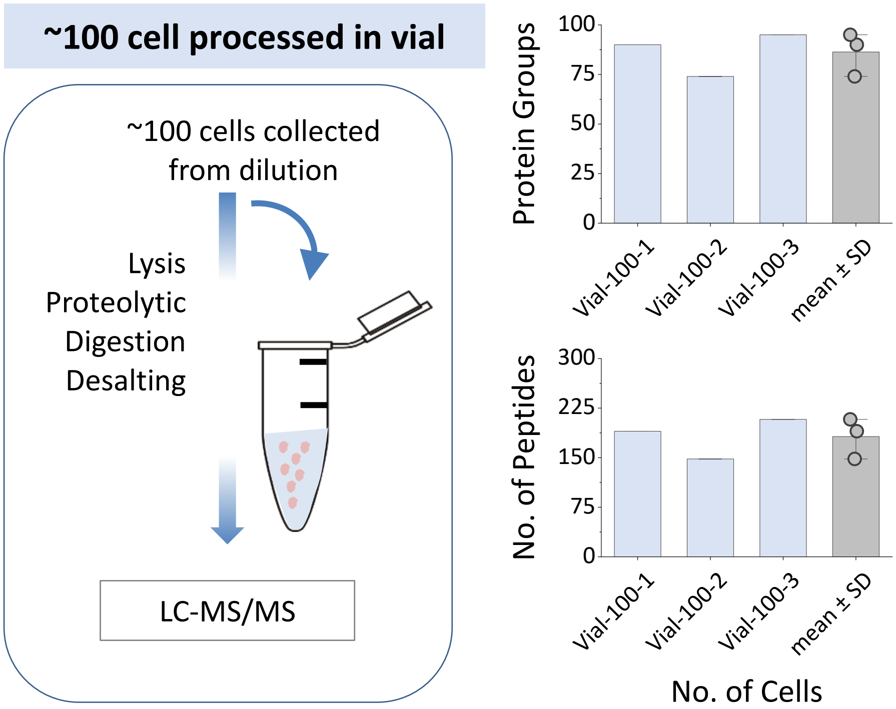

Supplementary Figure 13. Processing of ~100 cells in vial-based preparation. The number of proteins and peptides identified across a triplicate of 100-cell samples processed through vial-based preparation workflow. Data are presented as mean values ± SD (*n* = 3 independent experiments). Source data are provided as a Source Data file.

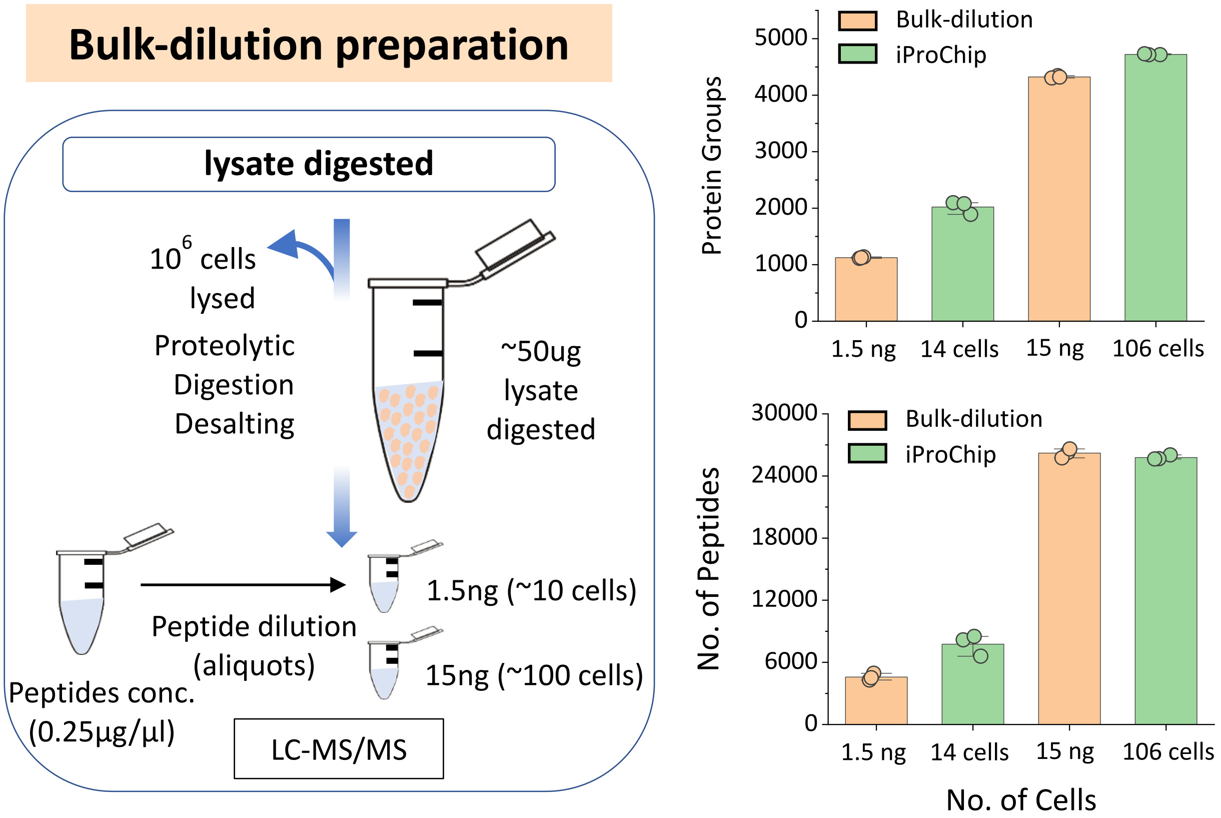

Supplementary Figure 14. Processing of 1.5 ng and 15 ng peptide samples through bulk-dilution processing and proteome coverage comparison. The number of proteins and peptides identified across the average of triplicates processed through bulk-dilution samples and their comparison with 14 cells and 106 cells processed by iProChip platform. Data are presented as mean values ± SD (*n* = 3 independent experiment). Source data are provided as a Source Data file.

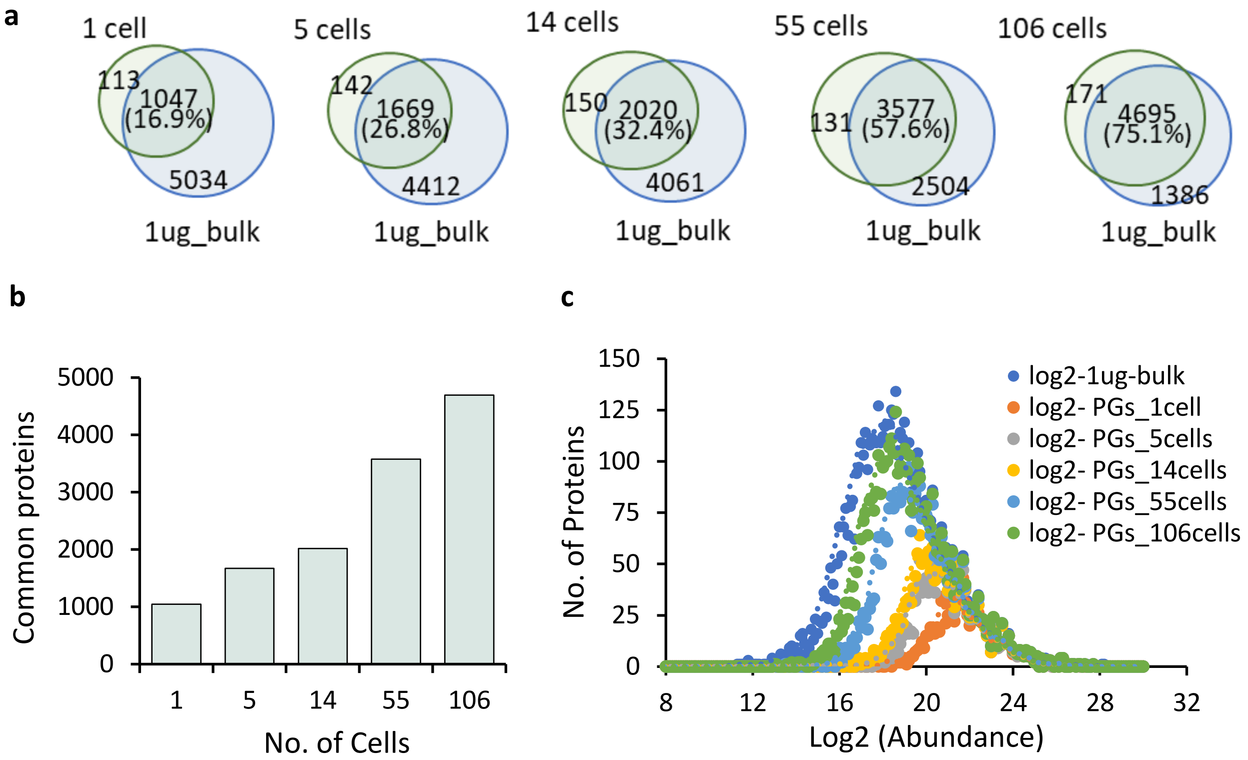

Supplementary Figure 15. Comparison of triplicate analyses results obtained from iProChip (1-100 cells) and bulk 1ug sample preparation. (a) Venn diagram showing proteins commonly identified from bulk sample preparation and iProChip. (b) Number (fraction) of proteins identified in bulk sample preparation that were also captured in the low-cells-number assay using iProChip. (c) Comparison of protein abundance distribution in commonly overlapped proteins among low-cells-data and bulk analysis. Source data are provided as a Source Data file.

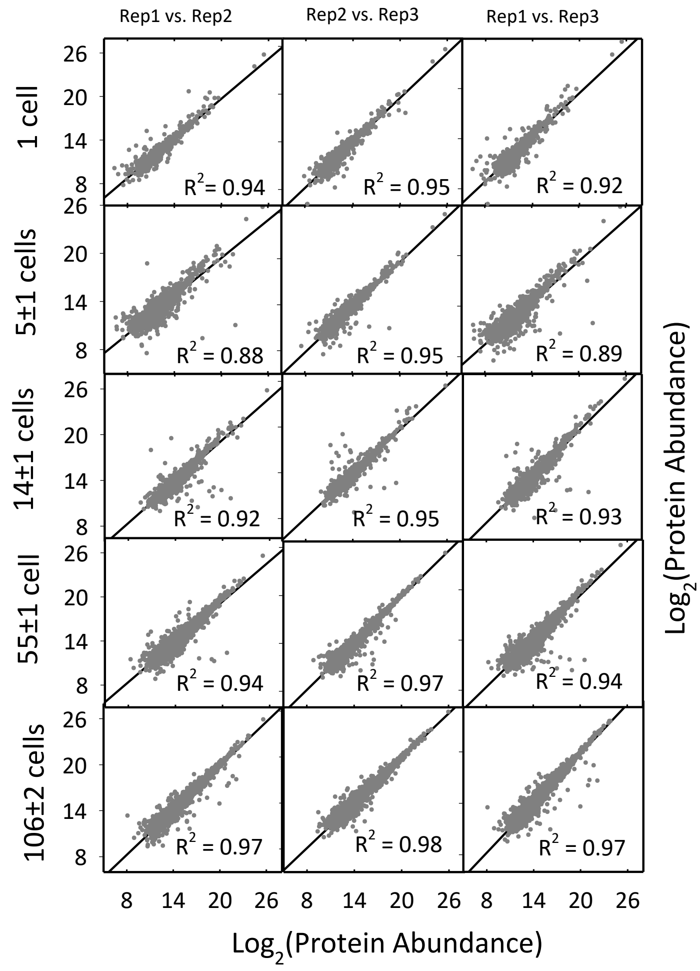

Supplementary Figure 16. Assessment of quantification reproducibility in triplicate analysis of PC-9 cells. Pairwise Pearson's correlation was calculated for protein abundances of all protein groups quantified in triplicate analysis among different numbers of PC-9 cells. Source data are provided as a Source Data file.

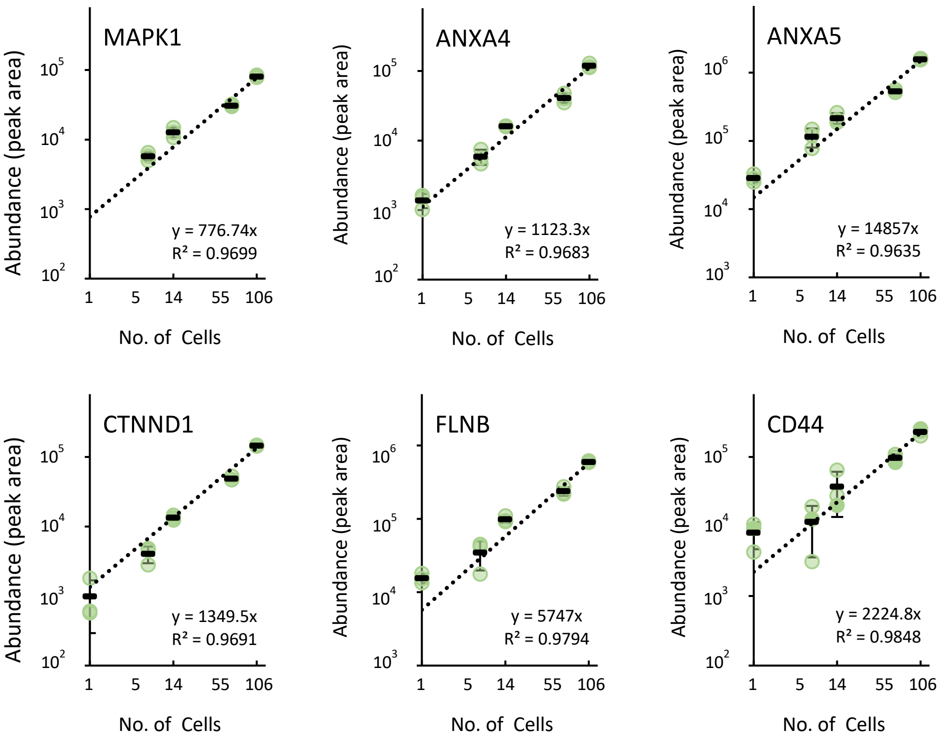

Supplementary Figure 17. Representative examples of lung cancer related proteins on quantitation of protein abundance in PC-9 cells. Additional examples of lung cancer related proteins on quantitation of protein abundance calculated from peak area. Data are presented as mean values ± SD (*n* = 3 independent experiment). Source data are provided as a Source Data file.

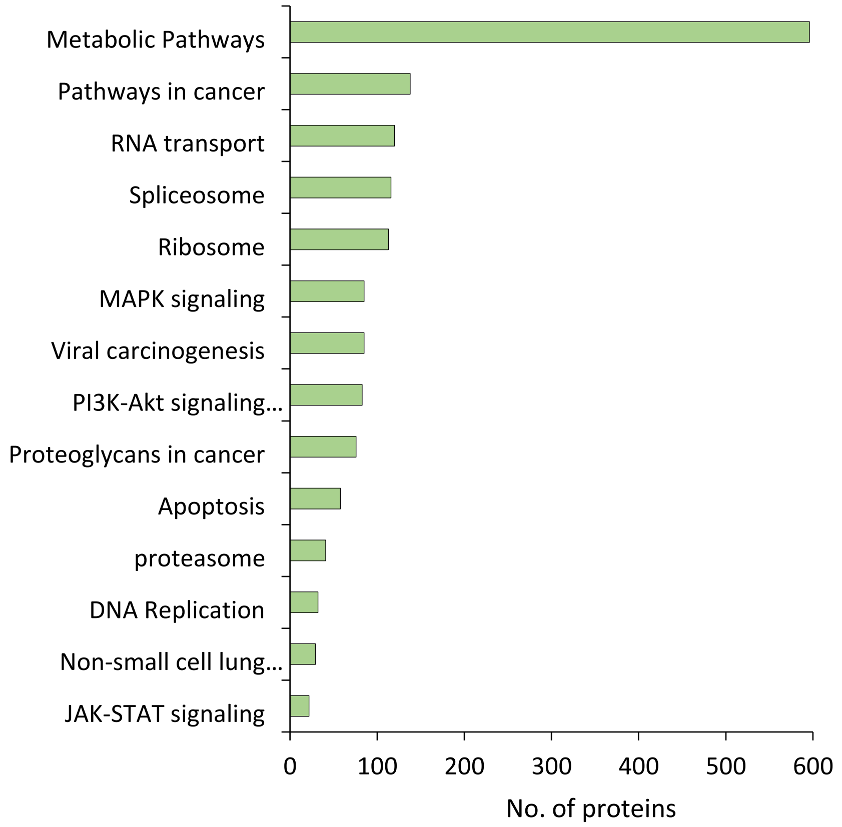

Supplementary Figure 18. Enrichment of pathways from protein identified in PC-9 cells by iProChip/DIA-MS analysis. A summary showing cancer-related pathways enriched from identified proteins in PC-9 cells using iProChip/DIA-MS analysis. Source data are provided as a Source Data file.

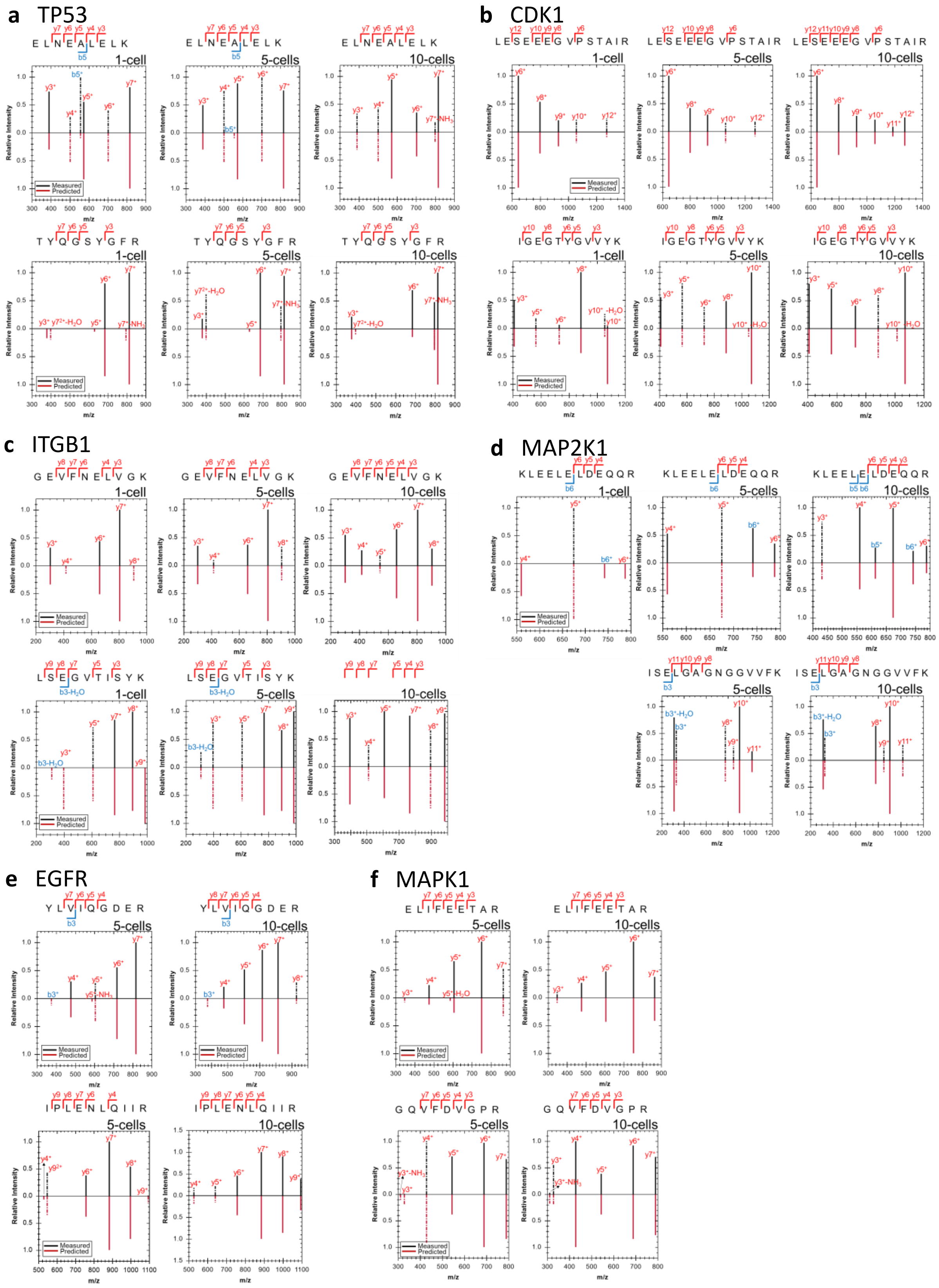

Supplementary Figure 19. Annotated MS2 spectra for the indicated peptides of proteins identified in 1-cell, 5-cell, and 10-cells of non-small lung cancer PC-9 cells, respectively. The corresponding peptide Q-value and quantity are shown in the supplementary table 3.

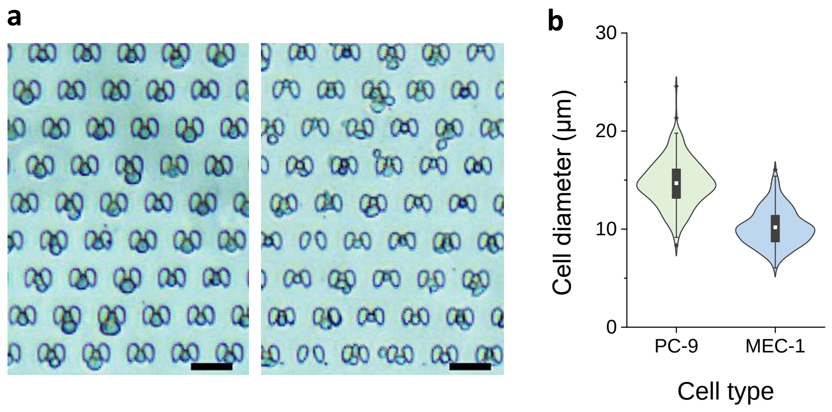

Supplementary Figure 20. Measurement of cell size of PC-9 and MEC-1 cells. (**a**) Images of captured PC-9 cells (left) and MEC-1 cells (right). Scale bars: 200 µm. (**b**) In our experiments, the mean cell diameter of PC-9 and MEC-1 cells were found to be 14.68 µm and 10.16 µm respectively. Each data was analyzed in n = 250 independent measurements. Center lines show the mean; box limits indicate the 25th and 75th percentiles; whiskers, 1.5× interquartile range. Source data are provided as a Source Data file.

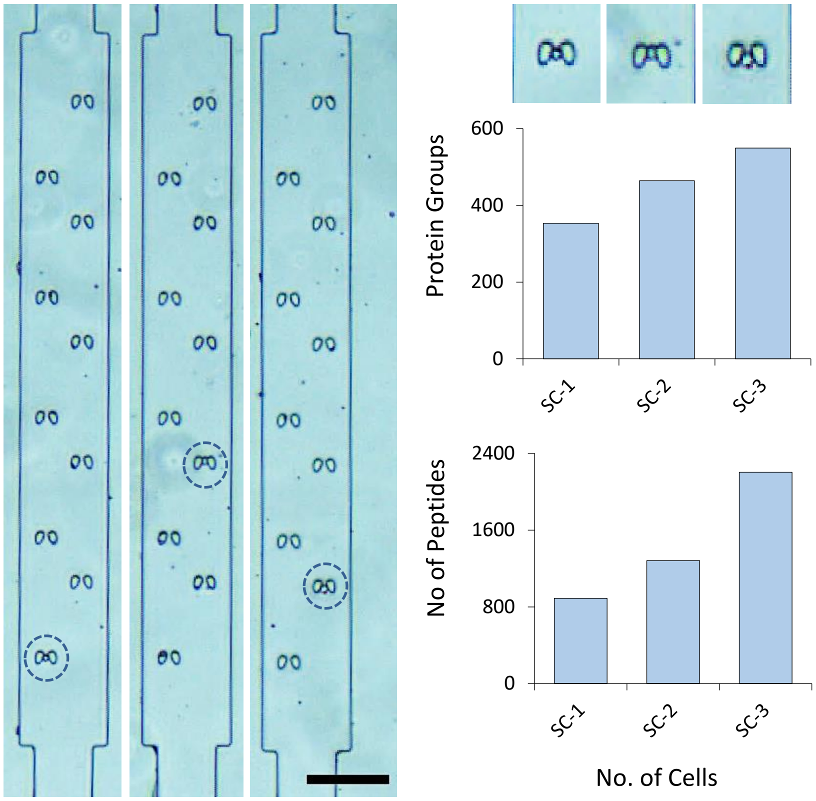

Supplementary Figure 21. iProChip-DIA processing of single MEC-1 cells. Trapping of 3 single MEC-1 cell (encircled) using the 10-cell capturing chamber for iProChip analysis. Individual cell image, corresponding protein groups, and peptides identified are shown. Scale bar: 150 µm. Source data are provided as a Source Data file.

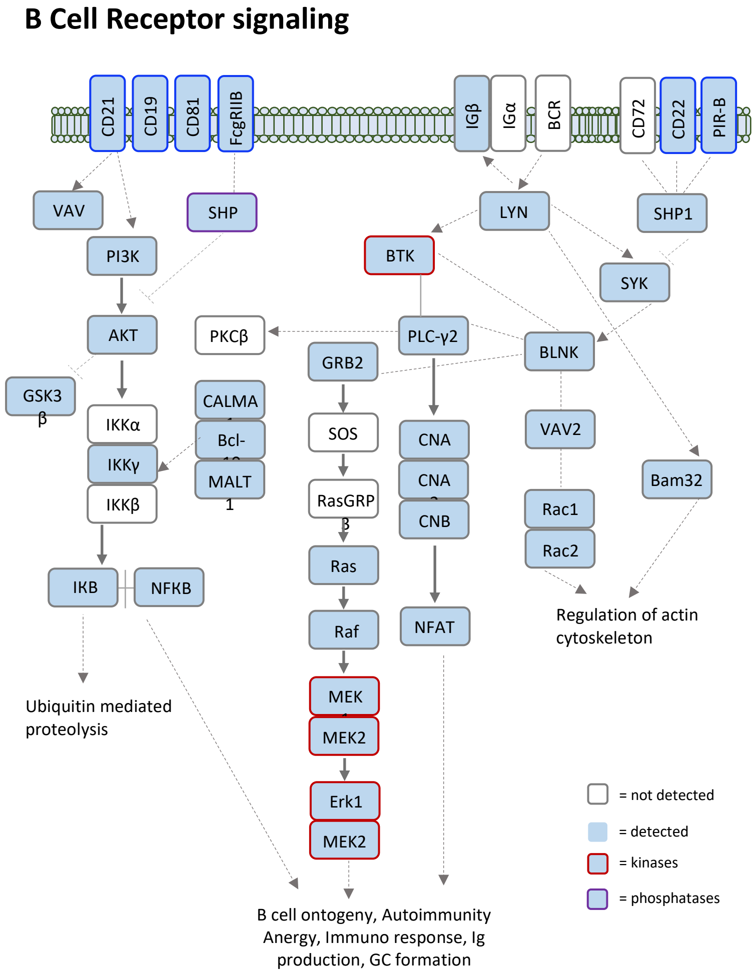

Supporting Figure 22. Identification of proteins related to B-cell receptor signaling pathway from combined results of 1-117 MEC-1 cells. The iProChip/DIA-MS analysis of 1-117 MEC-1 cells identified 43 out of 53 proteins in the B cell receptor signaling pathway. Source data are provided as a Source Data file.

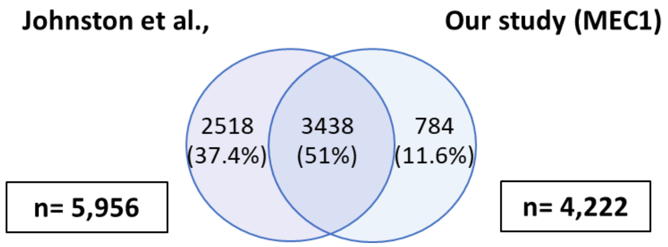

Supporting Figure 23. Venn diagram showing the overlap of commonly identified proteins among Johnston et al., study and the MEC-1 data identified in this study. Source data are provided as a Source Data file.

Supplementary Table 1. Surface area and volume in iProChip, SciProChip and nanoPOTS.

Device	iProChip					SciProChip			nanoPOTS
Module	Cell capturing chamber			Digestion chamber	Connecting channel*	Cell capturing chamber	Digestion chamber	Connecting channel *	well
No. cells	10	50	100	-	-	1	-	-	1
Volume (nL)	5	8.5	11	312	1.8	2.1	78.5	1.8	200
Surface area (mm^2)	0.46	0.81	6.4	6.9	0.18	0.2	6.4	0.18	0.8
Surface-to-volume ratio	0.09	0.09	0.02	0.02	0.1	0.09	0.08	0.1	0.004

** Connecting channel refers to the connection channel between the cell capturing chamber and the digestion vessel.*

Supplementary Table 2. Characterization of cell usage efficiency and input cells needed for operating the iProChip for 1, 5, 10, 50 and 100 cells.

Cell density (cells/µL)	Operational Volume (µL)	Flow Time (s)	No. of cells in operational volume[a]	Injected volume (µL)[b]	No. of cells injected	No. of cells trapped[c]	Cell usage efficiency (%)
25	5	3 ± 1	125	1 ± 0.2	25 ± 06	1 ± 0	4 ± 1.00
25	5	12 ± 3	125	4 ± 1.0	100 ± 25	5 ± 1	5 ± 0.26
25	10	24 ± 7	250	5 ± 1.0	125 ± 25	9 ± 1	8 ± 0.46
25	10	39 ± 7	250	6 ± 2.0	150 ± 50	50 ± 3	33 ± 7.32
25	10	36 ± 3	250	9 ± 1.0	225 ± 25	100 ± 4	44 ± 2.51

a. *Operational volume is the volume of cell solution for running the chip.*
b. *Injected volume is determined by measuring the remaining volume of cell solution in the vial.*
c. *Cell usage efficiency is defined as (number of trapped cells/number of total injected cells) x 100%*

Supplementary Table 3. Summary of peptide Q-value and quantity for the indicated proteins identified in 1-cell, 5-cells, and 10-cells of non-small lung cancer PC-9 cells, respectively.

Protein Groups	Peptide Groups	Peptide Qvalue			Peptide Quantity		
		**1-cell**	**5-cells**	**10-cells**	**1-cell**	**5-cells**	**10-cells**
**TP53**							
	ELNEALELK	5.4E-03	2.1E-29	2.1E-69	1.1E+00	6.8E+03	9.6E+03
	KPLDGEYFTLQIR	N/D	5.9E-12	2.7E-08	N/D	3.8E+03	4.6E+03
	LGFLHSGTAK	N/D	2.5E-04	6.1E-03	N/D	7.6E+02	1.1E+03
	TYQGSYGFR	2.3E-04	6.8E-19	5.6E-15	2.5E+03	6.4E+03	6.5E+03
**CDK1**							
	IGEGTYGVVYK	3.2E-04	2.4E-12	4.2E-29	5.6E+02	2.3E+03	5.4E+03
	LESEEEGVPSTAIR	8.6E-42	2.6E-25	8.6E-84	5.4E+03	1.3E+04	3.6E+04
	MLIYDPAK	N/D	N/D	7.0E-03	N/D	N/D	8.2E+03
	NLDENGLDLLSK	3.7E-04	9.1E-19	3.8E-57	5.6E+02	1.8E+03	1.3E+04
	SPEVLLGSAR	N/D	6.3E-05	9.8E-72	N/D	3.0E+04	4.1E+04
**ITGB1**							
	GEVFNELVGK	2.6E-15	9.3E-29	1.1E-56	2.2E+03	1.1E+04	2.1E+04
	LKPEDITQIQPQQLVLR	N/D	1.6E-13	1.1E-42	N/D	8.2E+02	1.5E+04
	LSEGVTISYK	3.5E-03	1.7E-19	2.0E-46	6.9E+02	5.6E+03	1.3E+04
	NVLSLTNK	1.8E-03	7.0E-16	3.3E-03	4.8E+02	5.8E+03	7.8E+03
**MAP2K1**							
	DVKPSNILVNSR	N/D	3.8E-04	4.2E-27	N/D	2.1E+03	5.8E+03
	ISELGAGNGGVVFK	N/D	5.7E-11	1.0E-58	N/D	6.6E+03	1.1E+04
	KLEELELDEQQR	1.5E-03	1.6E-17	1.9E-39	4.5E+02	6.4E+03	8.2E+03
	LEAFLTQK	N/D	3.7E-04	6.8E-29	N/D	2.4E+03	2.8E+03
	RLEAFLTQK	N/D	5.9E-05	N/D	N/D	1.5E+03	N/D
**EGFR**							
	EISDGDVIISGNK	N/D	6.2E-03	1.3E-43	N/D	7.8E+02	6.2E+03
	IPLENLQIIR	N/D	1.5E-07	3.2E-33	N/D	2.9E+03	1.4E+04
	VLGSGAFGTVYK	N/D	3.4E-04	2.3E-16	N/D	9.7E+02	3.2E+03
	YLVIQGDER	N/D	9.3E-09	1.8E-13	N/D	4.9E+03	8.1E+03
	RPAGSVQNPVYHNQPLNPAPSR	N/D	N/D	2.7E-05	N/D	N/D	3.9E+02
**MAPK1**							
	ALDLLDK	N/D	N/D	9.6E-03	N/D	N/D	4.5E+02
	ELIFEETAR	N/D	2.8E-09	3.6E-30	N/D	9.1E+03	2.0E+04
	GQVFDVGPR	N/D	4.2E-05	3.2E-07	N/D	4.0E+03	7.9E+03
	NYLLSLPHK	N/D	N/D	7.5E-03	N/D	N/D	2.8E+03

Note: N/D means "Not Detected"

Supplementary Table 4. Comparison of the identification of B-cell surface markers and important NSCLC proteins among datasets from PC-9, MEC-1 and B-cell leukemia cells.

Cells	Key Proteins	Accession Number	Our study (PC-9)	Our study (MEC-1)	B-cell Leukemia cells[1]
PC9	EGFR	P00533	✓	X	X
	TP53	P04637	✓	X	X
	MAP2K1	Q02750	✓	✓	✓
	MAPK1	P28482	✓	✓	✓
	CDK1	P06493	✓	✓	✓
	ITGB1	P05556	✓	X	✓
	PGK1	P00558	✓	✓	✓
	CDK4	P11802	✓	✓	X
MEC-1	CD19	P15391	X	✓	✓
	CD20	P11836	X	✓	✓
	CD21	P20023	X	✓	✓
	CD22	P20273	X	✓	✓
	CD81	P60033	✓	✓	X
	CD47	Q08722	✓	✓	✓
	CD74	P04233	X	✓	✓
	HLA-DRA	P01903	X	✓	✓
	HLA-DRB5	Q30154	X	✓	✓

Supplementary References:

1. Johnston, H.E. et al. Proteomics Profiling of CLL Versus Healthy B-cells Identifies Putative Therapeutic Targets and a Subtype-independent Signature of Spliceosome Dysregulation. *Mol Cell Proteomics* **17**, 776-791 (2018).
