# Supplementary material for: Streamlined single-cell proteomics by an integrated microfluidic chip and data-independent acquisition mass spectrometry
Source: Nat Commun. 2022 Jan 10;13:37. doi: 10.1038/s41467-021-27778-4 (PMC8748772; doi:10.1038/s41467-021-27778-4)
Supplement: Supplementary file 3 — Description of Additional Supplementary Files [file 41467_2021_27778_MOESM3_ESM.pdf]

Description of Additional Supplementary Files

File Name: Supplementary Data 1

Description: Proteomic dataset of PC-9 cells processed using the iProChip-DIA workflow.

File Name: Supplementary Data 2

Description: Proteomic dataset of MEC-1 cells processed using the iProChip-DIA workflow.

File Name: Supplementary Data 3

Description: Proteomic dataset of PC-9 cells processed using the SciProChip-DIA workflow.

File Name: Supplementary Movie 1

Description: Time-lapse video demonstrating packing of the solid phase extraction column by suspension of reverse phase C 18 beads in acetone. Scale bar: 600 µm.

File Name: Supplementary Movie 2

Description: Time-lapse video showing capturing of nonsmall lung cancer PC-9 cells by 10, 50 and 100 cell capture chambers. Scale bar: 150 µm.

File Name: Supplementary Movie 3

Description: Sample preparation steps in iProChip; cell capturing and lysis. Scale bar: 150 µm.
