# Supplementary material for: Streamlined single-cell proteomics by an integrated microfluidic chip and data-independent acquisition mass spectrometry
Source: Nat Commun. 2022 Jan 10;13:37. doi: 10.1038/s41467-021-27778-4 (PMC8748772; doi:10.1038/s41467-021-27778-4)
Supplement: Supplementary file 10 — Reporting Summary [file 41467_2021_27778_MOESM10_ESM.pdf]

nature portfolio

Corresponding author(s): Hsiung-Lin Tu, Yu-Ju Chen

Last updated by author(s): 2021/11/15

Reporting Summary

Nature Portfolio wishes to improve the reproducibility of the work that we publish. This form provides structure for consistency and transparency in reporting. For further information on Nature Portfolio policies, see our Editorial Policies and the Editorial Policy Checklist.

Statistics

For all statistical analyses, confirm that the following items are present in the figure legend, table legend, main text, or Methods section.

n/a	Confirmed	
[ ]	[x]	The exact sample size (*n*) for each experimental group/condition, given as a discrete number and unit of measurement
[ ]	[x]	A statement on whether measurements were taken from distinct samples or whether the same sample was measured repeatedly
[x]	[ ]	The statistical test(s) used AND whether they are one- or two-sided<br>*Only common tests should be described solely by name; describe more complex techniques in the Methods section.*
[ ]	[x]	A description of all covariates tested
[ ]	[x]	A description of any assumptions or corrections, such as tests of normality and adjustment for multiple comparisons
[ ]	[x]	A full description of the statistical parameters including central tendency (e.g. means) or other basic estimates (e.g. regression coefficient) AND variation (e.g. standard deviation) or associated estimates of uncertainty (e.g. confidence intervals)
[x]	[ ]	For null hypothesis testing, the test statistic (e.g. *F*, *t*, *r*) with confidence intervals, effect sizes, degrees of freedom and *P* value noted<br>*Give P values as exact values whenever suitable.*
[x]	[ ]	For Bayesian analysis, information on the choice of priors and Markov chain Monte Carlo settings
[x]	[ ]	For hierarchical and complex designs, identification of the appropriate level for tests and full reporting of outcomes
[ ]	[x]	Estimates of effect sizes (e.g. Cohen's *d*, Pearson's *r*), indicating how they were calculated

Our web collection on statistics for biologists contains articles on many of the points above.

Software and code

Policy information about availability of computer code

Data collection	Mass spectrometry data were collected using Orbitrap Eclipse Tribrid mass spectrometer (Thermo Fisher Scientific) coupled with an Ultimate 3000 RSLCnano system (Thermo Fisher Scientific) and Xcalibur (v 4.3.73.11) commercial software. A description about the code for chip control was included in the Code availability section, and is available at public repository Zenodo with a citable DOI.
Data analysis	MaxQuant (v1.5.6.5) software was used for DDA data analysis. Spectronaut (Biognosys, 13.11200127.43655) was used for spectral library construction and DIA data analysis. The data analyses and figures were drawn using Microsoft Office suit (2019), OriginPro (2021), SigmaPlot (10.0) and GraphPad Prism (v8.2.1). Cell size quantification and pixel intensities were computed by using ImagJ (v1.51).The pathway analyses were performed using the KEGG Mapper (2021, v05223) from the KEGG online database (https://www.kegg.jp/kegg/mapper.html). The kinome tree was drawn using KinMap (2021) online tool from Kinhub database platform (http://www.kinhub.org/kinmap).

For manuscripts utilizing custom algorithms or software that are central to the research but not yet described in published literature, software must be made available to editors and reviewers. We strongly encourage code deposition in a community repository (e.g. GitHub). See the Nature Portfolio guidelines for submitting code & software for further information.

Data

Policy information about availability of data

All manuscripts must include a data availability statement. This statement should provide the following information, where applicable:

- Accession codes, unique identifiers, or web links for publicly available datasets
- A description of any restrictions on data availability
- For clinical datasets or third party data, please ensure that the statement adheres to our policy

The mass spectrometry raw data sets, reference spectral libraries, and Spectronanut quantification outputs have been deposited in the Japan ProteOme Standard

Repository (jPOST; http://repository.jpostdb.org/); and can be accessed through ProteomeXchange (http://www.proteomexchange.org/) consortium. The dataset identifier is JPST000971 (https://repository.jpostdb.org/entry/JPST000971) for JPOST and PXD023325 (http://proteomecentral.proteomexchange.org/cgi/GetDataset?ID=PXD023325) for ProteomeXchange. The protein sequence fasta file was obtained from the UniProtKB human proteome database (https://www.uniprot.org/) and downloaded for "Homo Sapiens " (December, 2015). The iRT peptides fasta file was downloaded from Biognosys website (https://biognosys.com/product/irt-kit/#SupportMaterials). For pathways analysis and functional annotation, following databases were used: KEGG database (https://www.genome.jp/kegg/) and kinase families of KinMap database (http://www.kinhub.org/kinmap/). All data for iProChip/PC-9, iProChip/MEC-1 and SciProChip/PC-9 are available from the corresponding authors, and are provided herein as Supplementary Data 1, 2 and 3, respectively. All relevant source data are provided with this paper.

Field-specific reporting

Please select the one below that is the best fit for your research. If you are not sure, read the appropriate sections before making your selection.

☒ Life sciences ☐ Behavioural & social sciences ☐ Ecological, evolutionary & environmental sciences

For a reference copy of the document with all sections, see nature.com/documents/nr-reporting-summary-flat.pdf

Life sciences study design

All studies must disclose on these points even when the disclosure is negative.

Sample size	No statistical methods were used to predetermine the sample size in all experiments. A set of project specific spectra libraries (both at large-scale and small-scale) were constructed for lung cancer cell line (PC-9), and human chronic lymphocytic leukemia cell line (MEC-1) processed in iProChip or in vial-based processing. Both PC-9 and MEC-1 cell lines were used for DIA analysis. Technical or biological replicates of either three or ten were used. Samples size were chosen as multiple or triplicate measurements to ensure the quantitative reproducibility and demonstrate the DIA-based proteome analysis as routinely executed for method development studies.
Data exclusions	No data was excluded.
Replication	To demonstrate the pipeline for DA-based proteome analysis, experiments were performed multiple times, and most experiments were reproduced with at least three or ten replicates with similar results for the methodology development. Reproducibility was ensured using different cell lines.
Randomization	Not relevant to the study, as only cultured cells were used for method development. Only two cell line were used for the DIA analysis, randomization was not performed. For spectral library construction, randomization was not involved as all the datasets were merged and processed for peptide identification.
Blinding	Not relevant to the study. For DIA analysis, blinding was not performed as the focus is to demonstrate the technical performance of the workflow.

Reporting for specific materials, systems and methods

We require information from authors about some types of materials, experimental systems and methods used in many studies. Here, indicate whether each material, system or method listed is relevant to your study. If you are not sure if a list item applies to your research, read the appropriate section before selecting a response.

Materials & experimental systems

n/a	Involved in the study	
☒	☐	Antibodies
☐	☒	Eukaryotic cell lines
☒	☐	Palaeontology and archaeology
☒	☐	Animals and other organisms
☒	☐	Human research participants
☒	☐	Clinical data
☒	☐	Dual use research of concern

Methods

n/a	Involved in the study	
☒	☐	ChIP-seq
☒	☐	Flow cytometry
☒	☐	MRI-based neuroimaging

Eukaryotic cell lines

Policy information about cell lines

Cell line source(s)	The non-small cell lung cancer (NSCLC) cell line PC-9 was obtained from RIKEN BioResource Research Center (Japan, catalogue number: RCB4455). The human B-CLL cell line MEC-1 was a gift from Dr. Kuo I Lin (Genomic Research Centre, Academia SInica, Taiwan) and was originally obtained from DSMZ GmbH (Germany, catalogue number: ACC - 497).
Authentication	None of the cell lines were authenticated.
Mycoplasma contamination	The cell lines were tested negative for mycoplasm contamination.
Commonly misidentified lines (See ICLAC register)	No commonly misidentified cell lines are used in this study.
